# Postural adaptations to long-term training in Prader-Willi patients

**DOI:** 10.1186/1743-0003-8-26

**Published:** 2011-05-15

**Authors:** Paolo Capodaglio, Veronica Cimolin, Luca Vismara, Graziano Grugni, Cinzia Parisio, Olivia Sibilia, Manuela Galli

**Affiliations:** 1 Orthopaedic Rehabilitation Unit and Clinical Lab for Gait Analysis and Posture, Ospedale San Giuseppe, Istituto Auxologico Italiano, IRCCS, Via Cadorna 90, I-28824, Piancavallo (VB), Italy; 2 Bioeng. Dept., Politecnico di Milano, p.zza Leonardo Da Vinci 32, 20133, Milano, Italy; 3 Unit of Auxology, Ospedale San Giuseppe, Istituto Auxologico Italiano, IRCCS, Via Cadorna 90, I-28824, Piancavallo (VB), Italy; 4 IRCCS "San Raffaele Pisana", Tosinvest Sanità, Roma, Italy

## Abstract

**Background:**

Improving balance and reducing risk of falls is a relevant issue in Prader-Willi Syndrome (PWS). The present study aims to quantify the effect of a mixed training program on balance in patients with  PWS.

**Methods:**

Eleven adult PWS patients (mean age: 33.8 ± 4.3 years; mean BMI: 43.3 ± 5.9 Kg/m2) attended a 2-week training program including balance exercises during their hospital stay. At discharge, Group 1 (6 patients) continued the same exercises at home for 6 months, while Group 2 (5 patients) quitted the program. In both groups, a low-calorie, well-balanced diet of 1.200 kcal/day was advised. They were assessed at admission (PRE), after 2 weeks (POST1) and at 6-month (POST2). The assessment consisted of a clinical examination, video recording and 60-second postural evaluation on a force platform. Range of center of pressure (CoP) displacement in the antero-posterior direction (RANGE_AP _index) and the medio-lateral direction (RANGE_ML _index) and its total trajectory length were computed.

**Results:**

At POST1, no significant changes in all of the postural parameters were observed. At completion of the home program (POST2), the postural assessment did not reveal significant modifications. No changes in BMI were observed in PWS at POST2.

**Conclusions:**

Our results showed that a long-term mixed, but predominantly home-based training on PWS individuals was not effective in improving balance capacity. Possible causes of the lack of effectiveness of our intervention include lack of training specificity, an inadequate dose of exercise, an underestimation of the neural and sensory component in planning rehabilitation exercise and failed body weight reduction during the training. Also, the physiology of balance instability in these patients may possibly compose a complex puzzle not affected by our exercise training, mainly targeting muscle weakness.

## Background

Prader-Willi syndrome (PWS) is the most frequent cause of syndromic obesity and occurs in 1 in every 25,000 live births [1]. Its major clinical features include muscular hypotonia, childhood-onset obesity, short stature, small hands and feet, scoliosis, osteoporosis, hypogonadism and developmental delays [2]. Hyperphagia and weight gain between the ages of 1 and 6, lead most PWS patients to develop morbid obesity, affecting the development of motor and functional skills [3]. In adult life, although hypotonia does not progress, the progressive effects of obesity on the joints produce a cautious abnormal gait [4,5]. PWS patients present with reduced lean body mass and increased fat to lean mass ratio not only when compared with lean patients but also in relation to obese patients [6,7].

In general, obese individuals are typically sedentary as there is an inverse relationship between BMI and activity levels [8]. An increase in BMI is also associated with an increase in functional impairment [9], which could lead to impaired balance and an increased risk of falls than normal-weight individuals under daily postural stresses and perturbations [10,11], even in younger individuals, under 40 years of age [12,13]. Consequently, obese individuals may fear falling, which may lead to further reductions in physical activity [8], greater functional impairment [14], and greater risk of falling. Obesity associated with PWS is often massive and many individuals exceed by more than 200% their ideal body weight. In addition to that and muscular hypotonia, PWS show dysmorphic features that can affect postural stability, as short stature, small hands and feet, scoliosis and in fact they show a poorer balance capacity than their non-genetically obese counterparts [15]. It is therefore not surprisingly that fracture risk is approximately 50% in children [16] and more than 30% in adults [17]. The issue of whether rehabilitation interventions may improve balance and decrease risk of fall in PWS appears therefore certainly clinically relevant.

In a previous study [15], we demonstrated that PWS patients have a poorer balance capacity than their non-genetically obese counterparts and our findings suggested that strengthening of ankle flexors/extensors, balance training and tailored exercises aimed at improving medial-lateral control using hip strategies should be given particular consideration within rehabilitation programs.

Benefits from specific posture programs designed to improve balance and strength have been documented in obese patients [18], and weight reduction programs have a favorable impact on posture instability [13]. Maffiuletti et al [12] investigated the effect of a 3-week weight reduction program plus specific balance training on postural stability in extremely obese individuals. They demonstrated that a weight reduction program associated with a specific balance training was significantly more effective than the first alone. To our knowledge, no studies have quantitatively evaluated the effects of a training program on balance in PWS patients.

Vismara et al [19] have demonstrated that long-term group interventions (6 months) are feasible in PWS, despite their particular psychological profile, and effective in improving muscle strength and gait strategy.

The aim of this investigation was therefore to evaluate the effectiveness of a mixed exercise program, partially supervised and partially home-based, on postural stability in PWS adults.

## Methods

### Participants

We enrolled 11 adult patients with PWS (age: 33.8 ± 4.3 years; BMI: 43.3 ± 5.9 kg/m2) admitted to our rehabilitation hospital. Physical examination included determination of height and weight under fasting conditions and after voiding. BMI was defined as weight/height^2 ^(kg/m^2^). All patients showed the typical PWS clinical phenotype [20]. Cytogenetic analysis was performed in all participants; 10 had interstitial deletion of the proximal long arm of chromosome 15 (del15q11-q13). Moreover, uniparental maternal disomy for chromosome 15 (UPD15) was found in 1 female.

All PWS subjects showed mild mental retardation. One of the admission criteria for the study was a score over the cut-off value of 24 in the Mini Mental State Examination (MMSE) Italian version [21]. Scores over the MMSE cut-off suggest the absence of widespread acquired cognitive disorders in adult people. Our PWS patients were all able to understand and complete the testing.

The control group included 20 healthy individuals (CG: 10 females and 10 males; BMI: 21.6 ± 1.6 kg/m2; age: 30.5 ± 5.3 years). All participants were free from conditions associated with impaired balance. We clinically examined the experimental subjects to exclude individuals with vision loss/alteration, vestibular impairments, neuropathy and those who reported symptoms related to intracranial hypertension. All PWS and CG had normal values in the main laboratory tests, including adrenal and thyroid function. The study was approved by the Ethics Research Committee of the Institute. Written informed consent was obtained by patients, where applicable or their parents.

### Intervention

On admission, all patients underwent a clinical assessment. During their hospital stay, they attended a 2-week training program which included supervised exercise sessions with specific muscle strengthening of the lower limbs and 30-45 min aerobic walking sessions. All these sessions were held 4 days per week and included an introductory talk aimed at educating patients about the obesity-related changes in gait and posture and at providing practical information about their rehabilitation program. The sessions consisted of 4 exercises, explained as follows:

1) "Stand up against the wall without letting your heels touch it, bend at your knees to 90° as if you were about to sit down, then slowly return to the upright position";

2) "Stand up against the wall and then alternately lift your toes upwards";

3) "From a standing position, raise yourself up onto your toes and then slowly lower your heels back to the ground";

4) "Walk on your heels at a comfortable speed and don't let the rest of your feet touch the floor".

For exercises 1, 2, and 3 patients were asked to complete 3 sets of 15 repetitions each. For exercise 4, patients were asked to walk approximately 4 metres and then repeat the task 10 times with a rest in-between.

They were instructed to perform the exercise program at home 3 times a week for 6 months. Patients were required to keep a daily record of their adherence to the program.

At discharge, Group 1 (6 patients) continued the same 4 exercises unsupervised at home for 6 months, while Group 2 (5 patients) did not undergo the training program, according to a deliberate experimental design. In all subjects, a low-calorie, well-balanced diet of 1.200 kcal/day was advised during hospital stay and the 6-month home-training. Historically, the calorie requirement to maintain weight in adults with PWS is about 60% of normal, and a low calorie, well-balanced diet of 1,000-1,200 kcal/day combined with regular exercise should be advised [22]. Furthermore, a general recommendation to obtain weight loss has been from 800 to 1.000 kcal/day. In this light, adherence to these calorie-restricted diets requires intensive and continuous monitoring of intake by caregivers and regular dietary counselling.

### Methods

Subjects were assessed on admission (PRE), at discharge after 2 weeks (POST1) and after the 6-month training program (POST2). The assessment consisted of a clinical examination, video recording and postural evaluation.

The postural evaluation was conducted with a force platform (Kistler, CH; acquisition frequency: 500 Hz) integrated with a video system. The output of the force platform are three orthogonal components of ground reaction force (F_ML_, i.e. the component of ground reaction force in the medio-lateral direction, F_AP_, i.e. the component of ground reaction force in the antero-posterior direction; F_V_, i.e. the component of ground reaction force in vertical direction), a torsion moment, and the coordinate of Centre of Pressure (CoP) (CoP_ML_, i.e. the component of CoP displacement in the M/L direction and CoP_AP_, i.e. the component of CoP displacement in the A/P direction) on the horizontal plane.

The individuals were instructed to maintain an upright standing position for 60 seconds with open eyes (OE) focusing on a 6 cm black circle positioned at the individual line of vision at a distance of 1.5 m. Arms were hanging by their sides and feet were positioned at an angle of 30° with respect to the A/P direction. To standardize the experimental position, a triangle was located between the feet and removed just before acquisition. To avoid any kind of learning or fatigue effect [23] only one trial was acquired in this study for each session.

### Data analysis

The outputs of the force platform allowed us to compute the CoP time series in the A/P direction (CoP_AP_) and the M/L direction (CoP_ML_). The first 10s interval was discarded in order to avoid the transition phase in reaching the postural steady state [24].

In accordance with the literature [11] the following parameters were computed as significant for the postural analysis:

• RANGE: the range of CoP displacement in the A/P direction (RANGE_AP _index) and the M/L direction (RANGE_ML _index), expressed in mm;

• Sway Path (SP): the total CoP trajectory length, expressed in mm.

All parameters were normalized to the participant's height (expressed in meters), according to literature [25], in order to avoid the influence of different subject's height on the results.

### Statistical analysis

All the previously defined parameters were computed for each participant and then the mean values and standard deviations of all indexes were calculated for each sessions in PWS and for CG. Data of all patients were compared using Wilcoxon matched pair test, to detect significant PRE-POST1 differences; the same test was used to compared POST1 and POST2 of Group 1 and Group 2, considering each group separately. PWS and CG data were compared with Mann-Whitney U tests. Null hypotheses were rejected when probabilities were below 0.05.

## Results

In Table I, mean and standard deviation values for each postural parameter are displayed at PRE and POST1 for PWS and CG. The reported values were normalised for individual height (expressed in meters).

At PRE, the analysed parameters were statistically different in PWS and CG, suggesting that PWS patients did not present a physiological postural strategy. PWS individuals showed greater displacements along both the A/P and the M/L direction in terms of RANGE, in line with previous observations [12], and a longer SP than CG.

At POST1, no significant changes were observed in all of the parameters (Table 1) and BMI was similar to those observed at PRE session (43.04 ± 7.43 kg/m^2^).

**Table 1:** Postural parameters of PWS at PRE and POST1.

	*PRE*	*POST1*	*CG*
RANGE_AP_	19.04 (6.76)*	17.67 (5.24)*	5.03 (2.65)

RANGE_ML_	14.79 (9.53)*	12.59 (5.21)*	9.36 (3.53)

SP	573.58 (86.19)*	513.03 (80.90)*	201.33 (45.86)

Data are expressed as mean (standard deviation) (expressed in mm) and are normalised to the participant's height (expressed in meters). CG: Control group. * = p < 0.05, PRE and POST1 versus CG.

**Figure 1:**
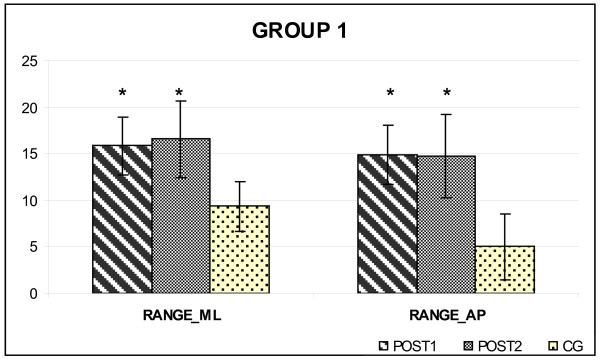
**Postural parameters of GROUP1 at POST1 and POST2**. Data are expressed as mean (standard deviation) (expressed in mm) and are normalised to the participant's height (expressed in meters). CG: Control group. * = p < 0.05, POST1 and POST2 versus CG.

**Figure 2:**
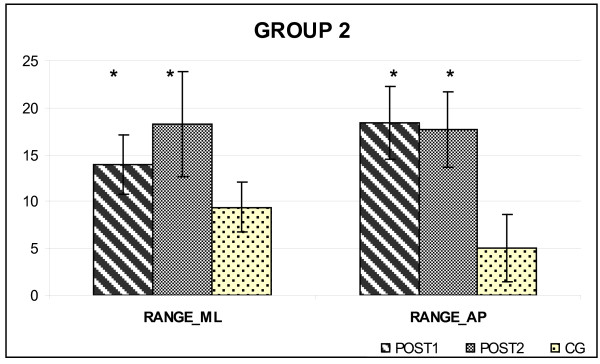
**Postural parameters of GROUP2 at POST1 and POST2**. Data are expressed as mean (standard deviation) (expressed in mm) and are normalised to the participant's height (expressed in meters). CG: Control group. * = p < 0.05, POST1 and POST2 versus CG.

At POST2, Group 1 (6 patients, undergoing the rehabilitative treatment at home for 6 months) and Group 2 (5 patients, non exercising after the completion of the 2-week supervised program) were compared. The adherence to the home-based program, computed as the percentage of number of sessions performed/number of total sessions, was 90%. The postural condition of both groups of PWS patients were unchanged (Figure 1, Figure 2), maintaining the higher values for RANGE_AP _and RANGE_ML _parameters. The SP did not differ significantly both in GROUP1 (498.74 ± 70.26 vs. 469.53 ± 58.67; p > 0.05) and in GROUP2 (527.32 ± 91-18 vs. 506.63 ± 90.92; p > 0.05), too.

At POST2, BMI was similar to those observed in basal condition both in GROUP1 (40.38 ± 3.46 kg/m^2 ^vs. 42.57 ± 4.92 kg/m^2^; p > 0.05) and in GROUP2 (42.54 ± 7.69 kg/m^2 ^vs. 38.35 ± 2.13 kg/m^2^; p > 0.05).

## Discussion

The issue of whether rehabilitation interventions may improve balance and decrease risk of fall in PWS certainly appears clinically relevant. PWS patients are characterized by an increase in BMI which is demonstrated to be associated with an increase in functional impairment [9], which could lead to impaired balance and an increased risk of falls. As obese individuals generally fear falling, further reductions in physical activity [8], greater functional impairment [14], and greater risk of falling may occur.

Based on a recent study of our group on PWS [19] showing that long-term strengthening and gait training is feasible and effective, with the present investigation we aimed to verify if the reduced balance could be amended by specific training. For PWS, providing an effective and simple home-based training would represent a continuum of the rehabilitation process outside the hospital which appears crucial in all chronic conditions. Given the psychological profile of PWS individuals, training sessions should be kept simple and reasonably short to guarantee compliance to the program. The exercises we prescribed were simple, clearly explained and did not exceed a total of 30 min/day. Also, patients had been previously familiarized with the exercises and supervised for 2 weeks in order to make sure they would be able to perform them properly at home in the following 6-month training period.

Our initial hypothesis was that balance is mainly reduced in PWS because of muscle weakness [7] and our training addressed the muscle groups that had been found to be mainly responsible for gait disorders [5]. In particular our training focused on ankle flexor and extensor muscle groups but did not include specific balance and proprioceptive exercises. A main reason for that was to provide simple and repetitive exercises, confined in a limited lapse of time, to be safely rehearsed by the patients at home. Adding diverse exercises might have jeopardized compliance in patients who psychologically need reassuring repetitive tasks.

Unfortunately, our results suggest that, unlike our previous positive results in strength gain and gait improvement [19] and despite a high adherence to the program, no postural adaptations occur after a long-term, mainly home-based strength training of the lower limb muscles. We have chosen this type of intervention on the basis of the known reduced muscle tonus and strength in PWS, which have been acknowledged as major causes of poorer balance.

A possible bias of our study is the relatively small sample size, although it should be reminded that PWS is a rare condition and large experimental groups are difficult to gather. As overweight is a distinctive feature in PWS, the analysis should have been more rigorously compared with obese instead of normal-weight individuals. However, the main object of our investigation was to assess quantitatively the effect of a mixed training program on balance in patients with PWS. Also, the low intensity of the home-based program may have played a role in the negative results. Although exercise intensity at home was not measured, anti-gravity resistance exercises in subjects with an excess in body mass should provide adequate exercise intensity for the aim of improving function. Intensities as low as 60% of the maximum voluntary contraction have indeed proved to be an effective stimulus for strength and function gain in elderly subjects [26]. Apart from muscle weakness, the control of stability and posture requires a complex interaction of both the musculoskeletal and neural system. Balance capacity is also secondary to body alignment and muscle tone. The first factor can in fact minimise the effect of gravitational forces while the second counteracts gravity. Postural tone is fine tuned, among other factors, by intrinsic stiffness of the muscles and neural drive. Sensory/perceptual processes, involving the organisation and integration of visual, vestibular and proprioceptive systems also play a role. It can be speculated that our exercise program may have lacked of specificity with regard to balance and oversimplified the functions to be trained, mainly targeting muscle strengthening and sensory-motor integration. Also, the dose of exercise, in terms of intensity and duration of the program, could represent a possible cause of the lack of effectiveness of the training.

Our results are in contrast with those obtained by Maffiuletti et al [12] on morbidly obese individuals. In their study, specific balance training, in addition to a body weight reduction program, improved significantly the postural strategy of these patients. In our study, training was indeed associated to the administration of a hypocaloric diet, but weight reduction is difficult to achieve in adults PWS due to their insatiable appetite and food-seeking behavior. It could be speculated that weight loss in addition to specific balance training is mandatory in order to improve balance capacity in PWS.

Baseline postural capacity is different in the two populations, with PWS patients generally characterized by poorer balance than their non-genetically counterparts. The mechanisms underlying this reduced capacity have not been thoroughly investigated in PWS. Future research will need to address quality and quantity of exercises targeted at improving balance capacity in PWS as well as to unveil the physiological determinants of instability in PWS.

## Conclusions

In this study we evaluated quantitatively the effectiveness of a mixed exercise program, partially supervised and partially home-based, on postural stability in PWS adults. Our results suggest that no postural adaptations occur after this program, unlike our previous positive results in strength gain and gait improvement and despite a high adherence to the program. Probably the low dose of intensity of exercise, in terms of intensity and duration of the program, associated to the lack of specific balance training may have played a role in the negative results and could represent a possible cause of the lack of effectiveness of the training.

These results are important from a clinical and rehabilitative point of view as they suggest the need of enhancing quality and quantity of exercises targeted at improving balance capacity in PWS patients.

## Competing interests

All authors haven't any conflicts of interest and any financial interest.

All authors attest and affirm that the material within has not been and will not be submitted for publication elsewhere

## Authors' contributions

PC made contribution to conception, design and interpretation of data, revising the manuscript critically and gave the final approval of the manuscript; VC made substantial contributions to analysis and interpretation of data and was involved in drafting the manuscript; LV made substantial contributions to data acquisition, elaboration and interpretation; GG made contribution to interpretation of data, revising the manuscript critically; CP made contribution to interpretation of data, revising the manuscript critically; OS made contribution to interpretation of data and to revision of the final version of the manuscript; MG made contribution to conception, design and interpretation of data, revising the manuscript critically and gave the final approval of the manuscript.

All authors have read and approved the final manuscript.
